# Primary hepatic neuroendocrine tumors: multi-modal imaging features with pathological correlations

**DOI:** 10.1186/s40644-017-0120-x

**Published:** 2017-07-06

**Authors:** Kai Yang, Ying-Sheng Cheng, Ji-Jin Yang, Xu Jiang, Ji-Xiang Guo

**Affiliations:** 1 Department of Radiology and Medical Imaging, Shanghai Sixth People’s Hospital, Shanghai Jiao Tong University, 600 Yi Shan Road, Shanghai, 200233 China; 2 Department of Interventional Radiology, Affiliated Changhai Hospital of Second Military Medical University, Shanghai, 200433 China

**Keywords:** Liver carcinoma, Neuroendocrine carcinoma, Computed tomography, Magnetic resonance imaging, Digital subtraction angiography

## Abstract

**Background:**

Primary hepatic neuroendocrine carcinomas (PHNECs) are rare and asymptomatic, and are therefore difficult to distinguish radiologically from other liver carcinomas. In this study, we aimed to determine the computed tomography (CT), magnetic resonance imaging (MRI), and digital subtraction angiography (DSA) features of PHNECs.

**Methods:**

A retrospective analysis of 11 patients with pathologically proven PHNECs was performed from January 2009 to September 2014. The CT, MRI, and DSA image features were analysed.

**Results:**

Ten of the eleven patients exhibited two or more lesions, and one patient exhibited a single lesion. Abdominal CT of 8 cases revealed multiple round or oval-shaped masses with well-defined borders, which were heterogeneous and hypodense on precontrast CT images. Significant diffuse heterogeneous enhancement was observed during the arterial phase in 8 cases, and the enhancement was slightly higher than the attenuation of the surrounding normal liver parenchyma and indistinct edges of small lesions during the portal phase. Well circumscribed (11 cases), lobulated (5 cases) or multiple nodular masses (4 cases), nodule (1 case) and irregular masses (1 case) of high signal intensity were observed on T2WI and DWI of MR images. The masses were well circumscribed, heterogeneous, and hypointense on T1WI, with significant enhancement of the solid carcinoma portion in the early arterial phase and continued enhancement in the portal venous phase. Characteristic lobulated or multiple nodular masses were observed in MRI. DSA showed multiple hypervascular carcinoma-staining lesions with sharp edges in the arterial phase.

**Conclusion:**

The CT, MRI, and DSA images of PHNECs exhibit specific characteristic features. Appropriate combinations of the available imaging modalities could therefore optimize the evaluation of patients with PHNECs.

## Background

Neuroendocrine carcinomas (NECs) mainly occur in organs of the bronchopulmonary or gastrointestinal tract, such as the pancreas, ileum, or appendix, but can occur in almost any organ including the bladder, prostate, rectum, stomach, bronchus, and biliary tree [1]. Although over 80% of NECs found in the liver are metastatic, primary hepatic neuroendocrine carcinomas (PHNECs) are very rare and difficult to distinguish radiologically. When an NEC is found in the liver, a diagnosis of extrahepatic metastatic carcinoma must first be eliminated [2]. The first case of PHNEC was reported by Edmondson in 1958 [3]. Fewer than 100 cases of PHNECs have since been reported in English literature [4], mostly as case reports. To our knowledge, comprehensive analyses of multi-modality imaging of PHNECs have rarely been reported.

The present study describes the presentations of eleven cases of PHNECs on computed tomography (CT), magnetic resonance (MRI), and digital subtraction angiography (DSA) images in detail. The objective is to determine whether multi-modality imaging techniques can improve the accuracy of PHNEC diagnosis.

## Methods

### Study design

A retrospective analysis of 11 patients with pathologically proven PHNECs was performed from January 2009 to September 2014 at two academic institutions. The median follow-up time was 26 months (12–56 months). Four patients were male and seven were female, with a mean age of 54 years (range 37–71 years). Ten patients exhibited multiple hepatic hemangiomas on ultrasound (US) examination, and had a 2–3-year follow-up and the lesions had being increased constantly, which prompted CT- or US-guided biopsy and histological diagnosis of PHNET. One patient exhibited a single liver mass associated with abdominal discomfort; hepatectomy was performed. None of the patients had a history of hepatitis B virus infection or liver cirrhosis, and laboratory examination results were negative for hepatitis B e-antigen, e-antibody, and core antibody. Liver and kidney functions were normal. Ten patients were negative for alpha-fetoprotein (AFP), carbohydrate antigen 19–9 (CA 19–9), carcinoembryonic antigen (CEA), and neuron-specific enolase (NSE), and one patient showed an AFP level of 11.04 ng/ml (normal, <7 ng/ml), CA 19–9 level of 42.5 U/ml (normal, <34 U/ml), and NSE level of 21.55 μg/L (normal, <17 μg/L). Because NEC was not suspected, the urine 5-hydroxyindoleacetic acid (5-HIAA) level and serum chromogranin A (CgA) level were not tested. CT, MRI, DSA, and digestive endoscopy were performed for diagnosis. At least 1 year of follow-up medical check (CT, MRI, or digestive endoscopy) revealed no NEC in the stomach, duodenum, colon, or rectum. Because multiple hepatic hemangiomas were suspected on US examination at 2–3-year follow-up, octreotide scan and/or PET-﻿CT were not performed at the areas of localization.

MR﻿ images were obtained using a 3 T scanner in a standard abdominal coil by using a liver-specific contrast agent (gadoxetic acid, Bayer Healthcare). Hepatic MRI protocol consisting of fast spin echo (FSE) T2-weighting (TR/TE, 3400/104), T1-weighting(TR/TE, 4.2/1.9), and DWI with b-values of 800 and 200 s/mm^2^. Explorations with dynamic Liver Acquisition with Volume Acceleration (LAVA) in gradient-echo mode were achieved before and after injection of 0.2 ml/kg of gadoxetic acid. The standard hepatic MRI protocol used in all patients, this included axial T1-weighting with double echo in phase and T1-weighting GRE in phase and opposed phase, matrix size 416 × 256, slice thickness 4 mm, interslice gap of 0.5 mm, field of view 46 cm × 46 cm; the axial T2-weighting with single shot FSE and the T2-weighting using fast imaging employing steady state acquisition, the axial T2-weighting of FSE with fat suppression with respiratory gating; finally, LAVA in gradient echo mode was used before and after enhancement. Delay time is 5 minutes.

### Imaging analysis

All patients’ images were analysed by two chief radiologists blinded to the pathological findings. Imaging analysis included evaluation of carcinoma boundaries, size, location, presence or absence of calcification and cystic degeneration, and strengthened mode. Disagreements were settled by consultation and consensus.

### Pathology analysis

Histological sections of the 11 PHNECs were all prepared by 10% formaldehyde fixation and paraffin embedding, and were stained with hematoxylin and eosin (HE). All immunohistochemistry analysis was performed on sections prepared from cell blocks by using an automated immunostainer and a modified avidin–biotin peroxidase technique. The diagnosis was made by two senior pathologists in consensus.

## Results

### Carcinoma size and location

Of the 11 patients, ten (four male and six female patients) exhibited two or more lesions; the largest was located in the right liver lobe and was associated with multiple metastases. One patient (female) exhibited a single lesion in the right lobe. The masses ranged in size from 1.9 × 1.5 cm to 8.6 × 6.5 cm.

### Imaging findings (Table 1)

**Fig. 1:**
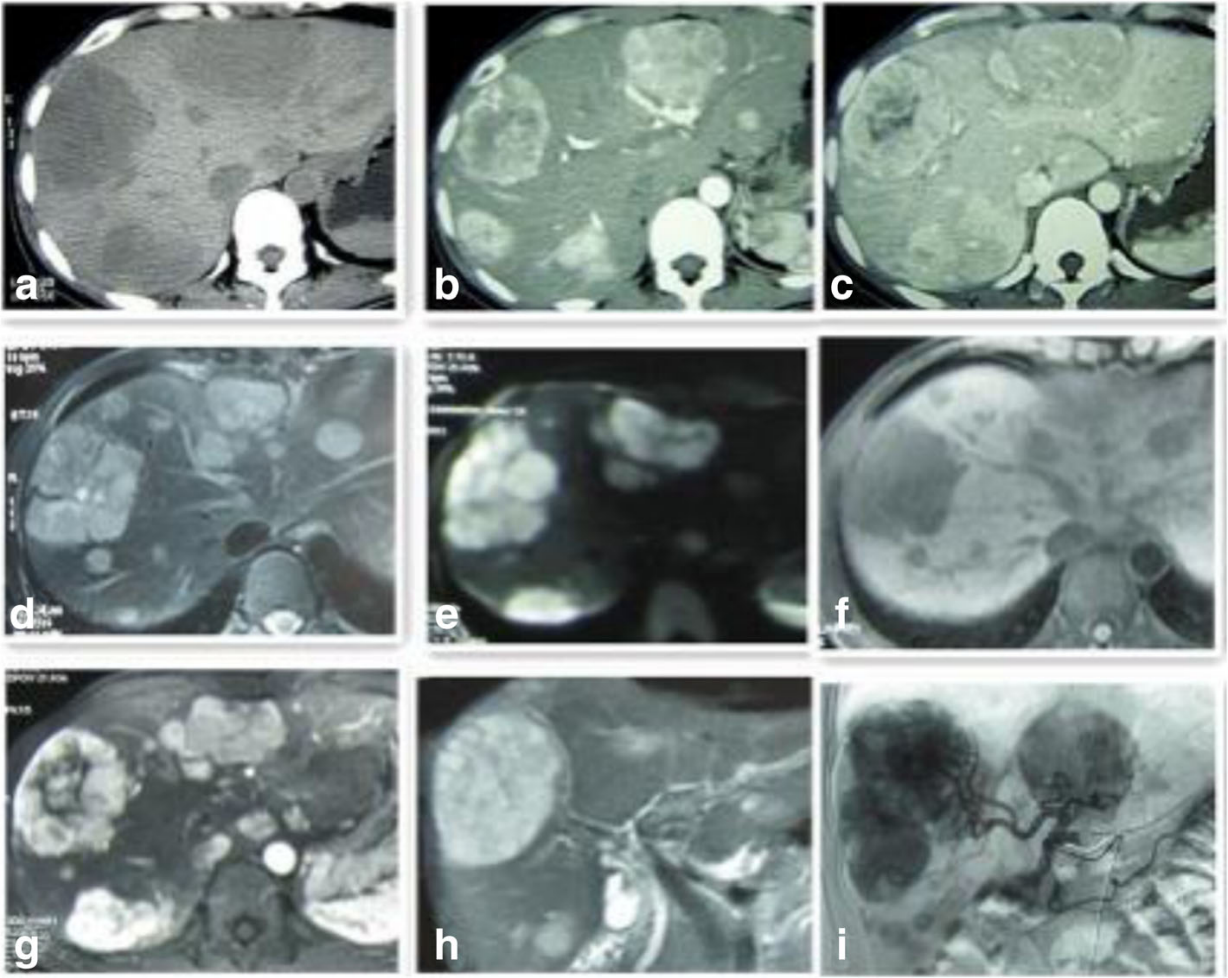
CT scan axial images (**a-c**). Multiple well-circumscribed, heterogeneous, and hypodense liver masses were observed (largest in the right lobe with size of 6.4×6.3×5.0 cm) (**a**), lesions showed significant enhancement in the arterial phase (**b**), and scope of enhancement was close to or slightly higher than the attenuation of the surrounding normal liver parenchyma and indistinct edge of small lesions during portal phases (**c**). The background liver was not cirrhotic. MR images (**d**-**i**). T2WI (**d**) and DWI (b = 800) ﻿(**e**): well-circumscribed, high signal intensity, lobulated masses were observed. Precontrast T1WI (**f**): lesions were well circumscribed and heterogeneous, and showed hypointensity. Enhanced MR images showed significant enhancement of the solid tumor portion in the early arterial phase, continued enhancement in the portal venous phase (**g**), definite defects in the 5 min delayed hepatobiliary phase, and well-rim arc-shaped artery vessels on the lesion side in the coronal image (**h**). DSA angiography: multiple hypervascular tumor staining regions with sharp edges were observed in the arterial phase (**i**)

**Fig. 2:**
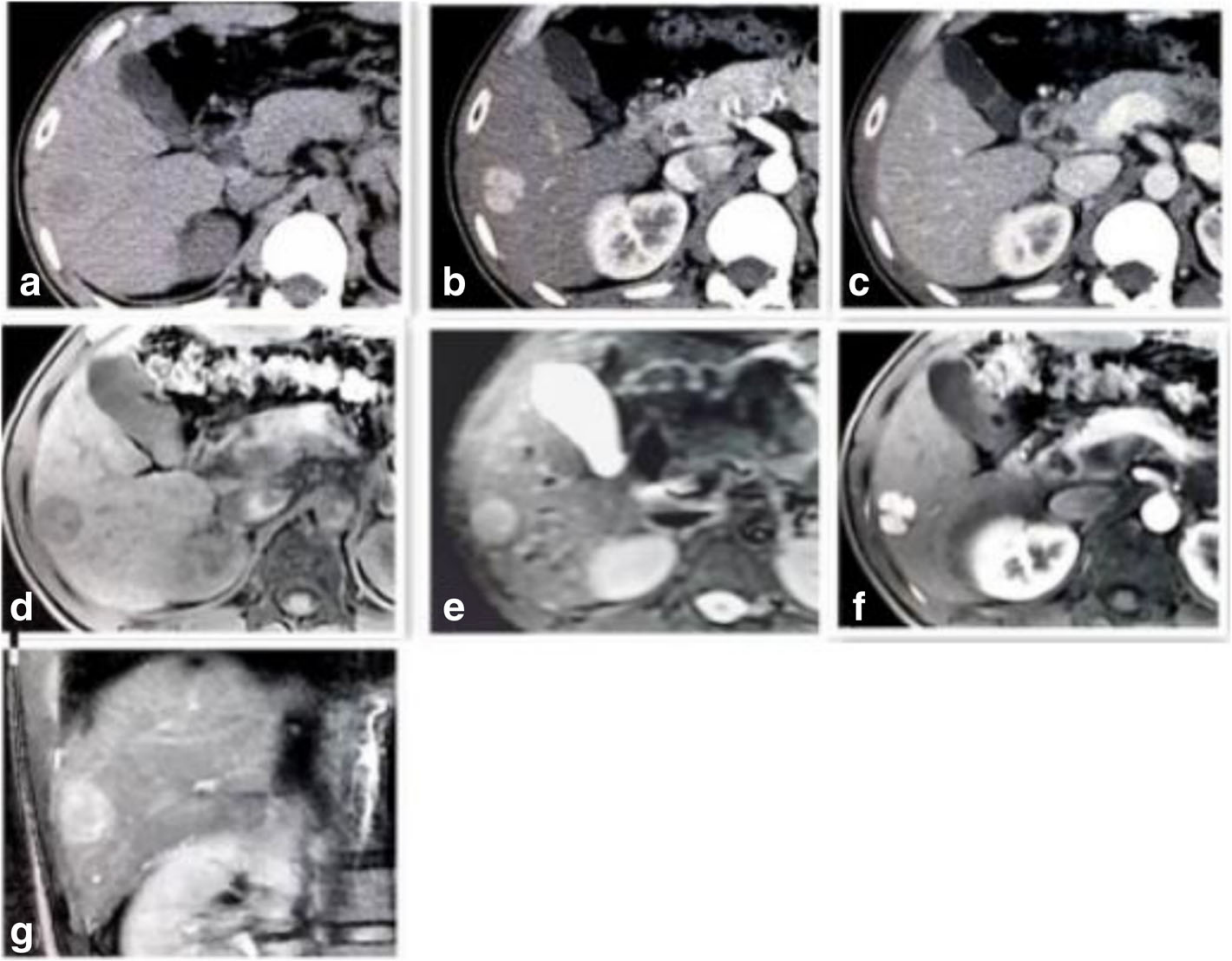
CT scan axial images (**a**-**c**) showing a hypodense liver mass in the right lobe, which demonstrated significant enhancement in the arterial phase and peripheral rim enhancement in the delayed phase. MR images (**d**-**g**), T1-weighted image (**d**): lesion shows well circumscribed, heterogeneous, and hypointense masses. T2-weighted axial image (**e**) shows well-circumscribed, high signal intensity masses. On enhanced MR images, lesions show significant enhancement and tumor lobulation in the early arterial phase (**f**), enhancement in the 5 min delayed image (**g**)

Abdominal CT of 8 cases revealed multiple round or oval-shaped masses with well-defined borders, which were heterogeneous and hypodense on precontrast CT images, ranging from 1.9 to 6.4 cm in diameter. During the arterial phase, all lesions showed significant diffuse heterogeneous enhancement. The scope of enhancement was close to or slightly higher than the attenuation of the surrounding normal liver parenchyma and indistinct edge of small lesions during the portal phase. One patient exhibited a pseudocapsule sign surrounding the lesion edge. The liver background was not cirrhotic. (Fig. 1a-c, Fig. 2a-c).

**Table 1:** Imaging Feature of 11 Cases of PHNEC

	CT					MRI								DSA
	unenhanced			enhancement		unenhanced					Gd-DTPA-enhanced			enhancement
	shape	border	density	Arterial phase	Venous phase	shape	border	T1WI	T2WI	DWI	Arterial phase	Venous phase	Delay phase	
case1	M-R-O	well	hypo	hetero	moderate	Lob	well	hypo	hyper	sig. Hyper	hetero	hyper	moderate	hyper
case2	M-R-O	well	hypo	hetero	moderate	Lob	well	hypo	hyper	sig. Hyper	hetero	moderate	mild	hyper
case3	M-R	well	hypo	hetero	moderate	Lob	well	hypo	hyper	hyper	hetero	moderate	mild	hyper
case4	M-R-O	well	hypo	hetero	moderate	Lob	well	hypo	hyper	sig. Hyper	hetero	hyper	moderate	hyper
case5	M-R-O	well	hypo	hetero	moderate	Lob	well	hypo	hyper	sig. Hyper	hetero	hyper	moderate	hyper
case6	M-O	well	hypo	hetero	moderate	M-N	well	hypo	hyper	sig. Hyper	hetero	moderate	mild	hyper
case7	M-O	well	hetero, hypo	hetero	moderate	M-N	well	hypo	hyper	sig. Hyper	hetero	moderate	mild	hyper
case8	S-R	well	hypo	homo	mild	nodule	well	hypo	hyper	hyper	homo	moderate	mild	hyper
case9						M-N	well	hypo	hyper	sig. Hyper	hetero	moderate	mild	hyper
case10						M-N	well	hypo	hyper	sig. Hyper	hetero	mild	mild	hyper
case11						Irr	well	hetero	hyper	hyper	hetero	hetero	hetero	hetero

*M* multiple, *S* single, *R* round, *O* oval, *well* well-defined, *hetero* heterogeneous, *hypo* hypodense, *hetero*, significant heterogeneous enhancement, *homo* significant homogeneous enhancement, *moderate* moderate enhancement, *mild* mild enhancement, *Lob* lobulated, *M-N* multiple nodule, *Irr* irregular, *well* well circumscribed, *hypo* hypointensity, *hyper* hyperintensity, *Sig* significant, *DAS*, *hyper* hypervascular.

**Fig. 3:**
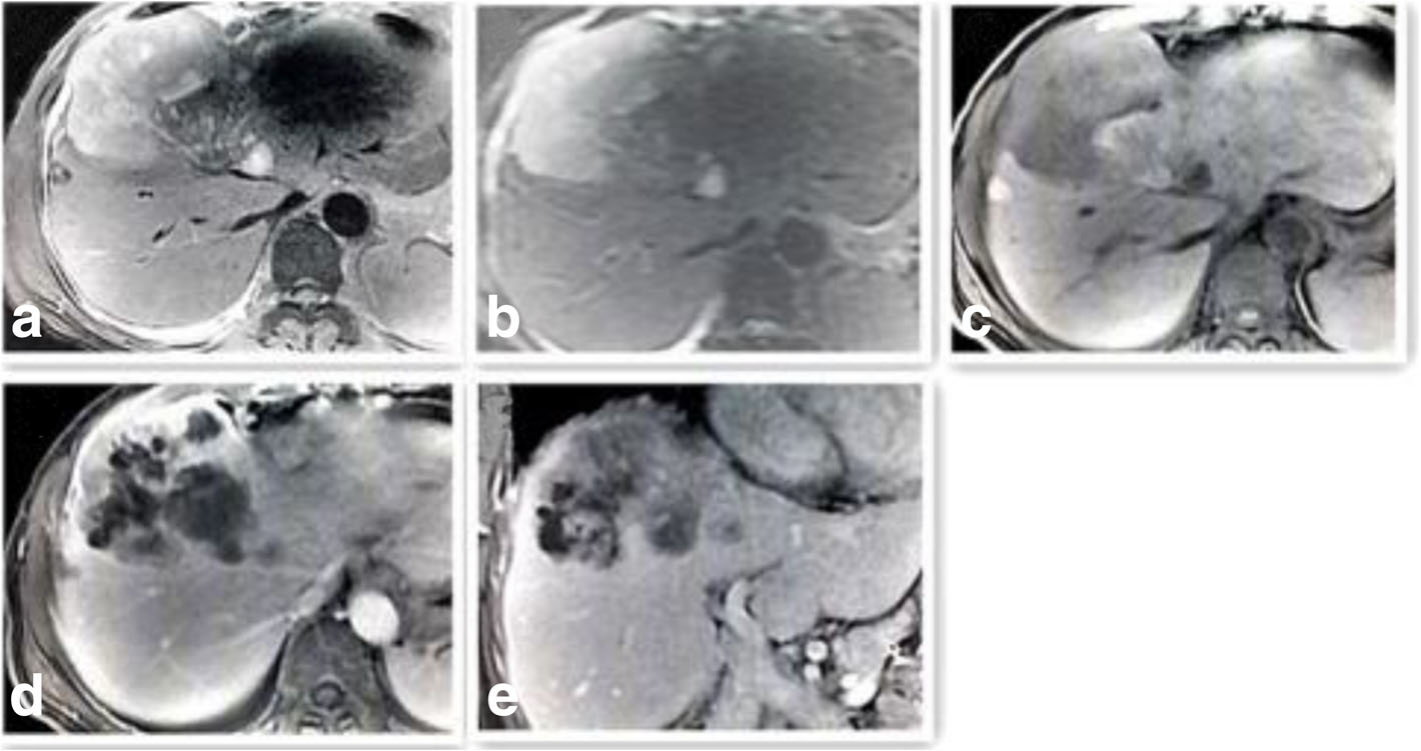
MR images (**a**-**e**). T2-weighted axial (**a**) and diffusion (b = 800) (**b**) images showing well-circumscribed areas, high signal intensity, and irregular large low signal intensity masses in the right liver lobe. T1-weighted image (**c**) showing well-circumscribed, heterogeneous, and hypointense masses. Irregular enhancement and no enhancement lumps were observed in the early arterial phase (**d**) and 5 min delayed hepatobiliary phase (**e**)

MR images: gadoxetic acid enhanced T1WI demonstrated significant nodular enhancement of the solid carcinoma portion in the early arterial phase, continued enhancement in the portal venous phase, and a high signal intensity or definite defect in the 20 min delayed hepatobiliary phase. Well circumscribed (11 cases), lobulated (5 cases) or multiple nodular masses (4 cases), nodule (1 case) and irregular masses (1 case) of high signal intensity were observed on T2WI and DWI of MR images. The masses were well circumscribed, heterogeneous, and hypointense on T1WI, with significant enhancement of the solid carcinoma portion in the early arterial phase and continued enhancement in the portal venous phase. Characteristic lobulated or multiple nodular masses were observed in MRI. T2WI and dDWI revealed well circumscribed lobulated or multiple nodular masses of high signal intensity. These masses were heterogeneous and hypointense (the largest was present in the right lobe and measured 6.4 × 6.5 × 5.1 cm) on T1WI. One patient exhibited well-rim arc-shaped arteries adjacent to the lesion in the coronal image. (Fig. 1d-i, Fig. 2d-g, Fig. 3a-e). Two patients exhibited hepatomegaly on CT and MRI imaging.

DSA angiography demonstrated multiple hypervascular carcinoma-staining lesions with sharp edges in the arterial phase (Fig. 1i ).

#### Pathological features

**Fig. 4:**
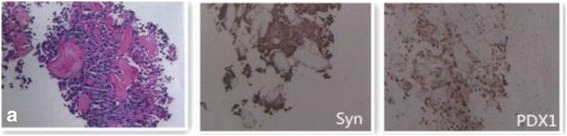
Microscopic findings in primary hepatic neuroendocrine tumors as observed under high magnification (200×). (**a**) The tumor cells appeared to surround a glass-like cylindrical arrangement of red stained substance (indicating cytoplasm); nuclei were counterstained. Immunohistochemical synaptophysin (**Syn**) staining and pancreatic and duodenal homeobox 1(**PDX1**) staining in positive tumor cells is also shown

Pathological examination revealed poorly, moderately, or mixed differentiated cells. The carcinoma cells were arranged in glandular tubes, trabeculae, irregular nests, or as a single structure. Nuclear atypia was obvious, and karyokinesis was present. The carcinoma cells surrounded a glass-like substance exhibiting a cylindrical arrangement; the cytoplasm was stained red, and the nucleus was darkly stained. (Fig. 4).

The results of immunohistochemical analysis are shown in Table 2.

**Table 2:** Results of immunohistochemical analysis

	case1	case2	case3	case4	case5	case6	case7	case8	case9	case 10	case 11
CAM5.2	+	+	+	+	+	+	+	+	+	+	+
Syn	+	+	+	+	+	+	+	+	+	+	+
PDX1	+	+	+	+	+	+	+	+	+	+	+
CK8/18	+	+	+	+	+	+	+	+	+	+	+
β-Tub	+	±	+	+	+	+	+	±	+	±	+
CD56	+	+	+	+	+	+	+	+	+	+	±
CD34	−	−	−	±	−	−	−	±	−	±	−
CD31	−	−	−	−	−	−	−	−	±	−	−
INHa	−	±	−	−	±	−	−	−	−	−	−
AFP	−	−	−	−	−	−	−	−	−	−	−
P53	−	−	−	−	−	−	−	−	−	−	−
Ki-67	5%	8%	4%	3%	10%	5%	6%	3%	7%	6%	4%
Hep	−	−	−	−	−	−	−	−	−	−	−

*CAM5.2* Cytokeratin CAM5.2, *Syn* synaptophysin, *PDX1* pancreatic and duodenal homeobox 1, *CK* cytokeratin, *β-Tub* β-Tublin, *INH* inhibin, *AFP* alpha-fetoprotein, *Hep* hepatitis

## Discussion

Neuroendocrine carcinomas (NECs) comprise only 1% to 2% of all gastrointestinal carcinomas. The most common site of NEC occurrence is the small intestine (45%); NECs have also been reported in the rectum (20%), appendix (17%), colon (11%), and stomach (7%) [1, 4]. With the extensive application of new technologies, the World Health Organization (WHO) found that it is more suitable to use the term “NEC” instead of “carcinoid carcinoma” [5], and updated the classification system in 2010, differentiating between the terms NEC and neuroendocrine carcinoma. Proliferation indices (Ki-67 and MIB-1), angioinvasion and mitoses are important factors for differentiation. NECs are divided into three main categories based on the malignancy potential of the carcinoma [6]: well-differentiated endocrine carcinoma (<2 cm in size and Ki-67 index of <2%, well-differentiated endocrine carcinoma (>2 cm in size, Ki-67 index of >2%, or presence of angioinvasion), and poorly differentiated endocrine carcinoma (Ki-67 index of >20%).

### Clinical features

PHNECs are very rare compared to the other NECs; however, there has been an increase in the incidence of these carcinomas over time [7]. PHNECs have been described as typically slow growing and non-functional in most case reports, occurring mainly in 40–50 year. Most PHNECs have been reported in female adults [5, 8]. In the present study, the mean age of the 11 patients was 54 years (range: 37–71 years), and the proportion of female patients was 63.7% (males: 36.3%). The right liver lobe appeared to be more commonly affected than the left lobe. This epidemiologic feature is concordant with our results. In the present study, masses were found in the left liver lobe in one patient and in the right lobe in seven patients. The relationship of PHNEC with hepatitis virus and cirrhosis remains unclear. None of the patients in the present study had a history of hepatitis B virus infection or liver cirrhosis. PHNECs do not therefore appear to be associated with underlying liver disease.

### Clinical symptoms

PHNECs may be found incidentally during routine screening. The most common symptoms found were distention or right upper quadrant pain, weight loss, and fatigue [9]. Carcinoid syndrome occurs in less than 10% of patients with gastrointestinal NECs, and is rare in patients with PHNECs [9]. No patients in the present study had carcinoid syndrome, and only one exhibited abdominal discomfort. Physical examination findings were also atypical. Only hepatomegaly was found in patients with advanced disease. Two patients exhibited hepatomegaly on CT and MR imaging.

### Laboratory tests

5-hydroxyindoleacetic acid (5-HIAA) in 24-hour urine specimens was performed with high specificity (90%) and low sensitivity (73%) [10]. The specificity of the serum chromogranin A ﻿(CgA) level ranges from 84% to 95%, and the sensitivity ranges from 87% to 100% [11]. However, CgA measurement may result in false-positive results in patients with hepatic and renal failure, atrophic gastritis, or chronic proton pump inhibitor use [12]. CgA can also be used to monitor carcinoma recurrence. The carcinoma markers CEA, CA19–9, and AFP are not specific for PHNECs. Because a diagnosis of NEC was not initially considered in the patients in the present study, the urine 5-HIAA level and serum CgA level were not tested in the preoperative period.

### Radiological imaging features

Based on radiological imaging, PHNECs can often be confused with other hepatic carcinomas. Thus, US, CT, and MRI have low sensitivity for the imaging of PHNECs [13]. US usually shows hypoechoic, hyperechoic, or mixed echogenic lesions with rings around them. Color US could lead to misdiagnosis as hemangioma because of bloodstream echo signals within the lesions. CT is the most frequently applied radiological technique to determine the localization of NECs and the prevalence of disease. In the present study, abdominal CT revealed multiple well circumscribed, heterogeneous, hypodense masses, and no lesions showed significant calcification. Significant diffuse heterogeneous enhancement was observed during the arterial phase and the scope of enhancement was close to or slightly higher than the attenuation of the surrounding normal liver parenchyma and indistinct edge of small lesions during the portal phases. The characteristics of the metastases and primary carcinomas were similar. MR images were obtained with a 3.0-T unit using a liver-specific contrast agent. Well-circumscribed, lobulated, or multiple nodular masses of high signal intensity were observed on T2WI and DWI of MR images. These masses were well circumscribed, heterogeneous, and hypointense on T1WI. Gadoxetic acid-enhanced T1WI demonstrated significant enhancement of the solid carcinoma portion in the early arterial phase, continued enhancement in the portal venous phase, and a high signal intensity or definite defect in the 5 min delayed hepatobiliary phase. The mechanism of this phenomenon is not clear. We think that maybe similar to the mechanism of hemangioma and one of the reason of misdiagnosis of US. DSA angiography demonstrated multiple hypervascular carcinoma staining lesions with sharp edges in the arterial phase.

Although the potential of positron emission tomography computed tomography (PET-CT) in the staging of NECs is not clear, PHNECs usually exhibit high 18F–fluoro-de-oxy-glucose (FDG) uptake [14]. Octreotide (somatostatin receptor analogue) scintigraphy (OctreoScan) is more effective in detecting the localization of the carcinoma than are other techniques. It has a sensitivity ranging from 85–90% [15]. In addition to determining the location of primary or recurrent tumors, another benefit of the OctreoScan is the ability to predict response to octreotide analogue therapy [16].

### Diagnosis and differential diagnosis

Differentiation between metastasis and PHNEC by using imaging analysis (US, CT, MR, DSA) or the results of histology and immunohistochemical analysis is difficult. When an NEC is found in the liver, extrahepatic metastatic carcinomas must first be excluded, so an octreotide scan and/or PET-CT should have been done for the localization of a possible PHNEC.

### Clinical treatment

Clinical treatments include surgical hepatectomy [17, 18], liver transplantation [19, 20], somatostatin analogues [21], transcatheter arterial chemoembolization (TACE) [22], radiofrequency ablation (RFA) [23] and chemotherapy.

## Conclusion

PHNECs are very rare and asymptomatic carcinomas, and are difficult to distinguish radiologically from other liver carcinomas. Our findings suggest that CT, MRI, and DSA images of PHNECs exhibit specific characteristic features, which could be used in combination to optimize the evaluation of patients with PHNECs.

## Abbreviations

AFP: Alpha-fetoprotein
CA 19–9: Carbohydrate antigen 19–9
CEA: Carcinoembryonic antigen
CT: Computed tomography
DSA: Digital subtraction angiography
MRI: Magnetic resonance imaging﻿
NSE: Neuron-specific enolase
PET-CT: Positron emission tomography computed tomography
PHNECs: Primary hepatic neuroendocrine carcinomas
RFA: Radiofrequency ablation
TACE: Transcatheter arterial chemoembolization
US: Ultrasound
